# How are African livestock farmers responding to climate variability and change? A systematic review

**DOI:** 10.1088/1748-9326/ae3591

**Published:** 2026-01-19

**Authors:** M A North, N B Hunter, K Queenan, R Slotow

**Affiliations:** 1 University of KwaZulu-Natal, Pietermaritzburg, South Africa; 2 Munster Technological University, Cork, Ireland; 3 Royal Veterinary College, London, United Kingdom

**Keywords:** adaptation, climate change, coping, evidence synthesis, research gaps, systematic map

## Abstract

Livestock underpin livelihoods and food security in Africa, using marginal lands to produce high-quality protein for the household or as a source of income. For mixed farmers, livestock provide draught power, manure for fields, and buffer variations in crop productivity. Livestock also hold cultural significance. However, African livestock farmers are especially vulnerable to climate hazards, and attempt to reduce impacts in different ways, with varied outcomes. A synthesis of the state of knowledge of livestock farmers’ responses to climate variability and change would assist policymakers and practitioners to make informed decisions, and guide researchers towards gaps that need to be filled. To that end, we systematically reviewed articles published between 2014 and 2022—the period between the end of the Intergovernmental Panel on Climate Change’s Fifth Assessment and the end of the Sixth, recording study metadata, farmers’ responses, and their drivers, outcomes, barriers and enablers. We included 186 articles from 32 countries (most frequently Kenya or Ethiopia), from which 1089 responses were coded for analysis. Responses by small-scale farmers of cattle, sheep and goats, typically as part of mixed crop-livestock systems, were most common (*n* = 816), with few documented responses of commercial farmers (121). Most responses pertained to changing herd management (437), followed by feed or pasture management (294). Drought was the most common climate driver of responses (567), while the most commonly mentioned barriers included financial constraints (116), a lack of knowledge or information (98), and government support (67). While there is a sizable body of literature on climate impacts and adaptation of livestock farmers, notable gaps included any work in Central and Northern Africa, and responses of commercial farming systems. In general, future research should focus on these gaps and on improving the depth of information collected, such as on barriers and enablers of adaptation, to better inform future interventions.

## 1. Introduction

The agricultural sector is vital for many African economies (NEPAD 2013), contributing, on average, 17% of African countries’ GDP (Valev *et al* 2025), and employing over half of workers in sub-Saharan Africa (Fox *et al* 2013, FAO and ECA 2018). Within this sector, livestock production contributes up to half of the African agricultural GDP (Simpkin *et al* 2020), while also serving as a vital safety net for agricultural or rural communities, helping households endure climate-related shocks (Thornton *et al* 2009). Edible livestock products, such as meat, milk, and eggs, are a rich source of highly bioavailable nutrients, essential amino acids and critical micronutrients (Schönfeldt *et al* 2013, Beal *et al* 2023), particularly important for children and pregnant women (Grace *et al* 2018). Equally, both edible and non-edible (hides, wool, manure) animal products serve as a source of household income, and animals themselves act as financial capital, available to be sold for cash in times of need (Bahta 2022). Moreover, livestock are inherently complementary to crop production, providing draught power (for ploughing fields and transport) and manure for improving soil health and fertility (Thornton 2010), while crop residues serve as feed during the post-harvest dry season (Eisler *et al* 2014). Finally, livestock, and cattle in particular, serve important socioeconomic functions in many African cultures, including for spiritual and ceremonial purposes, maintaining social and kinship ties, or as a form of non-monetary currency (Coertze 1986, Ainslie 2013, Shava and Masuku 2019).

However, the sector, and all the people depending on it, is highly vulnerable to the impacts of climate change (Makate 2019). Climate-related hazards like temperature extremes and unpredictable rainfall are well-known challenges for farmers, impacting livestock production both directly (such as through exposure to high temperatures) and indirectly (for example, where climate hazards reduce the availability of feed or increase the burden of parasites and diseases) (Thornton *et al* 2009, 2021, Rojas-Downing *et al* 2017, North *et al* 2023).

In Africa, climate change is already resulting in altered seasonal precipitation patterns and timing, an increase in the number of consecutive dry days, changes in total annual precipitation, more frequent and severe weather events, and increasing average temperatures (fewer cold days in winter, and more hot days with even higher temperatures than usual in summer) (Niang *et al* 2014, Hoegh-Guldberg *et al* 2018, Trisos *et al* 2022). Additionally, the combination of warmer temperatures and decreasing rainfall are leading to a reduction in soil moisture, which impacts plant growth (Hoegh-Guldberg *et al* 2018)—including in rangelands, and the production of feed for livestock (Niang *et al* 2014).

It is thus critical that farmers and governments implement strategic responses that anticipate these climate-related hazards and reduce their impacts, to improve the sustainability and resilience of livestock farmers across Africa. There are many recommended adaptations for farmers, including from the Food and Agriculture Organisation of the United Nations (FAO) (FAO 2013, 2019, Alvar-Beltrán *et al* 2021). However, despite broad agreement that the best way to support local-level adaptation is to start with, and build on, existing (indigenous) strategies (Niang *et al* 2014), there is currently a lack of systematic syntheses of the literature describing what African livestock farmers are already doing to cope with current conditions to inform this process. This makes it difficult to generalise policy-relevant recommendations, or identify adaptation projects worthy of receiving adaptation funding (Escarcha *et al* 2018, Ariom *et al* 2022).

The global discourse on adaptation has grown exponentially in the last few decades (Sietsma *et al* 2021), corresponding with an increasing focus on adaptation in the latest IPCC assessments, including the Fifth (AR5, published in 2013/2014) and Sixth Assessments (AR6, published in 2022/2023). With the IPCC assessment process described as one of the ‘most rigorous scientific assessment processes’ globally (Nalau *et al* 2024, p 1), the description of how African livestock farmers respond to climate variability and change in these reports may be regarded as a useful foundation on which this systematic review can build.

In the IPCC AR5 Working Group II (WGII) report, Chapter 9 (Rural Areas), while predominately focussing on crop-based agriculture, described the following responses of African livestock farmers: replacing cattle with hardier goats and camels; using mobility to make use of the spatial variability in resources; providing supplemental feed, using crop residues, or conserving feed; destocking by culling and sale; and developing improved livestock breeds (Dasgupta *et al* 2014). Chapter 22 (Africa) included two further examples: increasing use of boreholes and selection of heat-tolerant animals (Niang *et al* 2014).

During AR6, both the Special Report on Climate Change and Land and the WGII report touched on the adaptation of livestock farming systems (IPCC 2019, 2022). The high-level adaptation assessment in the Africa chapter showed that livestock management had less evidence available than crop and fisheries management, demonstrating a relative gap in the literature, as well as regional gaps, with Northern and Central Africa unrepresented in the literature (Trisos *et al* 2022).

There is, therefore, an imbalance in agricultural adaptation synthesis, with a strong focus on crop production, with few tangible examples of adaptation in African livestock-based agriculture. In addition, with the burgeoning body of adaptation literature challenging authors’ abilities to comprehensively review and assess the information available, particularly during AR6 (Callaghan *et al* 2020, Nalau *et al* 2024), there is increasing need for synthetic research papers to assist with this process (Minx *et al* 2017). Finally, the breadth of topics covered in IPCC reports and tight page limits, prevented these reports from presenting a comprehensive set of workable examples of adaptation responses that are specific to, and useful for, African livestock farmers. Such detail is what is required to inform policy development and resource allocations in Africa.

The aim of this study is therefore to systematically review what the literature says African farmers are doing to reduce the impacts of climate-related hazards on their livestock systems, focussing on papers published after AR5. We start by mapping what is known and where, and who is conducting the research; we then describe farmers’ responses, climate and non-climate drivers, and barriers and enablers that were reported. We also identify gaps in the literature and suggest areas and topics that need further study.

## 2. Methods

This review followed the ROSES framework for systematic evidence synthesis for tracking, recording and reporting the process (Haddaway *et al* 2017b).

### 2.1. Search strategy

Search terms and synonyms were compiled from key conceptual papers (conceptual papers included: Nienaber and Hahn (2007), Seo and Mendelsohn (2008), Ifejika Speranza (2010), Renaudeau *et al* (2012), and Mashizha (2019)), and arranged by the four components of the research question. These were: (1) African country names, (2) major livestock species and products, (3) hazards related to climate change and variability, and (4) actions associated with responding to climate hazards. These terms were tested in Scopus and Web of Science using iterative searches to identify a master list of search terms, with words being added or removed based on the relevance of the first few pages of search results as well as the total number of articles found by each search. Base terms and wildcards were combined to include all derivations of the search terms (for example, ‘alleviat*’ for alleviate or alleviating); similarly, some African country names were not included to shorten the search string (e.g. ‘Africa’ will also identify papers on ‘South Africa’). Google Translate was used to translate the English search terms into French to improve the language inclusivity of search results. The final search strings were selected based on: (1) ability to identify a large number of papers, (2) with minimal irrelevant results, and limited by (3) the maximum number of characters allowed by each citation index (supplementary information (SI) section 1).

Searches were conducted by tailoring the master list of terms for each of six separate citation indices (Scopus, Web of Science ‘All Databases’^4^4The University of KwaZulu-Natal (UKZN) subscription to Web of Science (July 2020) includes the following databases: Web of Science Core Collection (1945-present): Science Citation Index Expanded (1945-present), Social Sciences Citation Index (1956-present), Arts & Humanities Citation Index (1975-present), Conference Proceedings Citation Index–Science (2010-present), Conference Proceedings Citation Index–Social Science & Humanities (2010-present), Book Citation Index–Science (2010-present), Book Citation Index–Social Sciences & Humanities (2010-present), Emerging Sources Citation Index (2015-present); KCI-Korean Journal Database (1980-present); MEDLINE® (1950-present); Russian Science Citation Index (2005-present); SciELO Citation Index (2002-present)., CAB Direct, Sabinet, PubMed, and JSTOR). The main search, run in 2020, was followed by a secondary search in January 2023 to update the results to include articles published up until the end of 2022. Detailed terms for each database or platform are included in the SI. Where the citation index allowed, search terms were in English and French. Search results were not initially restricted by year of publication; however, due to resource limitations, during the initial screening process, all papers that were published before 2014 were excluded. A full distribution of search results by publication year is provided in figure SI.1.

### 2.2. Screening

Search results were screened sequentially by article titles, abstracts, and then full text PDFs, applying the inclusion and exclusion criteria listed in table SI.2. Screening was performed by the first author, with any ambiguous cases resolved during discussions with the second author. To avoid biasing against papers in local, less prestigious journals, and authors for whom English was not their mother tongue, the process of critical appraisal was very lightly applied, with only papers with severe faults (e.g. methodological flaws, factually incorrect background information presented, very poor writing, spelling errors throughout, or major problems with figures (including no axis labels, repeated figures) that suggest poor editorial and reviewer oversight) excluded. Full texts were obtained wherever possible—when behind paywalls, the authors were contacted by email or via ResearchGate, and unformatted copies stored on institutional websites were used for several papers. Articles that were not in English were translated using Google Translate before full-texts were screened.

### 2.3. Data extraction, coding, and analysis

The metadata of included papers (source type, journal name, publication date, and the country of first author affiliation), information on the study (country of study, livestock production system, livestock species, climate hazards being responded to (i.e. the ‘climate driver’)), and detail on all actions taken by farmers in response to climate hazards (including barriers, enabling conditions, and non-climate drivers) were extracted and recorded in Microsoft Excel.

Early during the review, it was noticed that very few papers mentioned compliance with ethical guidelines, or approval by research ethics committees/review boards. Since this is a critical part of ensuring research integrity and quality, particularly for research involving human participants (Gelling 1999), the authors decided to record whether included papers mention of ethical clearance as an additional research question.

Responses were then coded as follows: first, they were grouped into categories that emerged from and were refined during the review, noting the climate and non-climate drivers, the desired outcome, and barriers or enabling conditions; then, the responses were coded according to asset-based adaptation metrics (Frayne *et al* 2012) and adaptation process metrics (Thornton and Manasfi 2010). Barriers and enablers were categorised by drawing on key influential papers (Eisenack *et al* 2014, Sibiya *et al* 2023, Brullo *et al* 2024), and analysed by region and response type. Responses that were difficult to code—for example, where the writing of the original study lacked detail, or the response did not clearly fall within adaptation metric categories—were noted for discussion, and resolved, either through consensus or noting ‘could not be determined’ or by leaving that cell blank.

Some of the main coding variables and their explanations are presented in table 1. For a full list of the extracted data, coding categories, and explanations and examples, please see SI sections 4 and 5.

**Table 1.** Classification of the actors, livestock production systems, responses and barriers that were presented in the included articles.

Coding variables	Categories and explanation
**Studies**	

Actor type	- Individuals (i.e. farmers) - Communities - Organisation (government and non-governmental)

Farm scale^a^	- Small-scale (including subsistence or household producers) - Commercial (production primarily market-focused (Tibesigwa *et al* 2017), can vary in size)

Primary production system^b^	- Primarily crops (some livestock) - Mixed crop-livestock systems - Primarily livestock (some crops)

Farmer mobility^c^	- Sedentary - Nomadic/practice transhumance

Farmer tenure^b^	- Own land (private use) - Communal land

**Responses**	

Response category^c^	- Aid (including relief, receipt of money, feed, or other resources from government or NGOs) - Environmental management (responses intended to control degradation, bush encroachment or pests; e.g. burning) - Feed/pasture management (responses relating to feed or grazing resources) - Financial management (responses relating to financial resources, e.g. savings, access to credit) - Herd management (responses relating to the livestock) - Sales and marketing responses (relating to timing of sales or changes to prices) - Social responses (relating to social support networks, traditional institutions) - System change (e.g. livelihood diversification or change) - Water management (relating to water resources) - ‘Other’ (responses that do not fit into the other categories)

Barriers^d^	- *Lack of* land, water, feed or pasture, labour, knowledge or information, technologies, infrastructure, markets, finances or government support - *Presence of* pests/diseases, competition or conflict - *Relating to* social, cultural, traditional, procedural or administrative context

^a^ Modified from Queenan *et al* (2020): Justification of changes for this study: The included articles frequently did not provide sufficient information to distinguish scale at any finer detail, nor did the articles describe the farmers’ motivation for keeping livestock (profit or home consumption).

^b^ Modified from Escarcha *et al* (2018).

^c^ Derived during this study.

^d^ Categories informed by (Eisenack *et al* 2014, Sibiya *et al* 2023).

We first present the general patterns we observed in the literature at the level of the articles and responses, highlighting hotspots and knowledge gaps. We then describe characteristics of the different response categories, some or the more novel types of responses being implemented by African livestock farmers, and provide an overview of the more common barriers to and enablers of adaptation.

## 3. Results

### 3.1. Characteristics and description of the included articles

**Figure 1.**
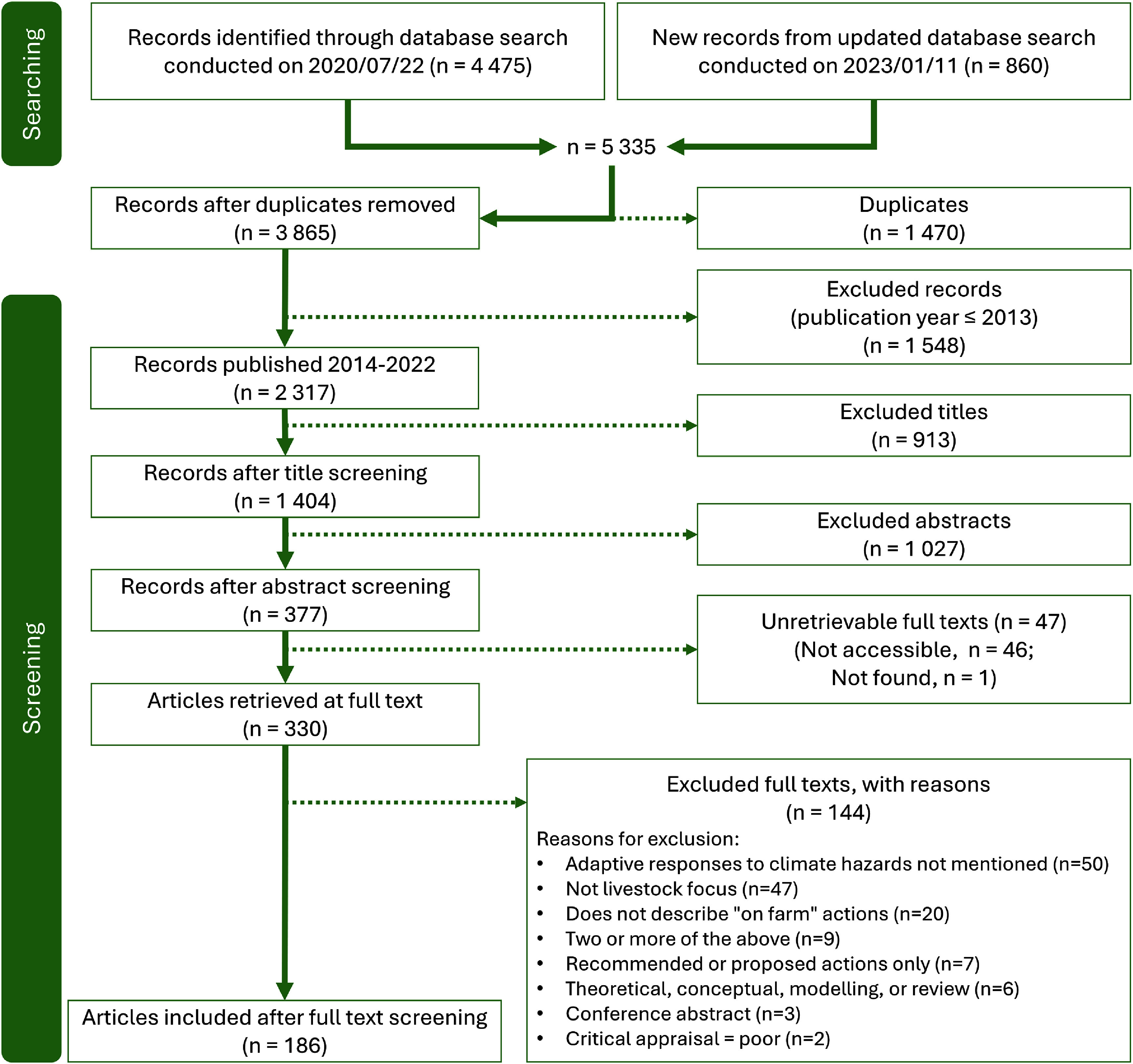
ROSES flow diagram of the results of the systematic review. Modified from Haddaway *et al* (2017a).

In all, 186 articles published between 1 January 2014 and 31 December 2022 were included (figure 1; for full list see SI). Most were peer-reviewed journal articles (*n* = 180 or 97%) published in 107 journals, and led by African authors (*n* = 152), primarily from Ethiopia, Kenya, or South Africa. Five of the included articles were in languages other than English: four in French, one in Japanese.

**Figure 2.**
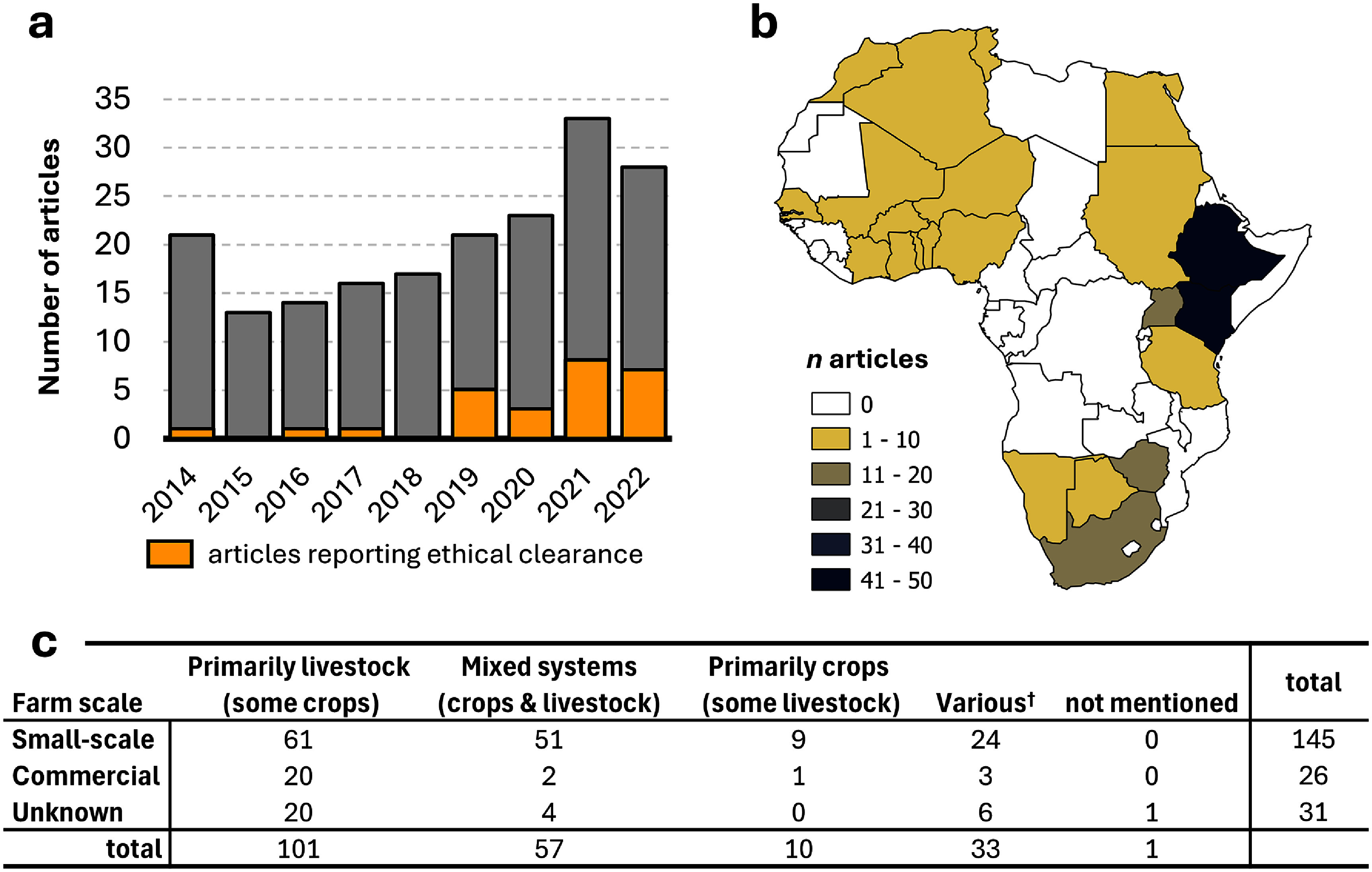
Overview of the included articles. (a) Number of articles that describe how African livestock farmers are responding to climate-related stressors per year between 2014–2022 (grey columns) and those that reported receiving ethical clearance (gold); (b) country where the research took place (*n* = 184; two articles covered multiple countries and were excluded from mapping); and (c) overview of the livestock systems represented in the included articles, with regard to scale and primary production system focus. ^†^ varied among the participants in the study.

While the number of articles published per year increased between 2014 and 2022, the increase was not as great as has been reported for other regions and topics relating to climate change (e.g. Berrang-Ford *et al* (2011), Callaghan *et al* (2020), and Hunter *et al* (2020); figure 2(a)). The relatively high number of articles on livestock adaptation published in 2014 may be the culmination of research conducted during, and in support of, the IPCC’s AR5 WGII report, which had a strong adaptation focus (IPCC 2014).

Only 26 articles (14%) reported obtaining ethical clearance or approval for their research, despite the fact that all these studies involved some form of stakeholder engagement (typically focus group discussions and key informant interviews). There was no clear pattern between reporting ethical clearance and first author affiliation, institution type or country; however, it appears that the number and proportion of articles reporting ethical approval is increasing over time (figure 2(a)). The lack of oversight by research ethics committees/institutional review boards may explain some weaknesses observed in study design. For example, many studies supplied participants with a closed list of responses to select from or rate (Oyekale 2014, Leal Filho *et al* 2017, Tetteh *et al* 2020, Ankrah Twumasi and Jiang 2021, Dika *et al* 2022). While facilitating statistical modelling, this approach precludes the identification of novel, potentially highly adaptive responses.

Another issue that better ethical oversight might have addressed was the over-research of some geographic areas, risking research fatigue in some communities, while other areas remain unstudied. For example, of the 42 articles based in Ethiopia, a country with 12 regional states, 11 took place in Oromia Regional State. In Kenya, a country with 47 counties, five of the 42 articles were based in Laikipia County, and four in Kajiado County.

Of the 55 countries located on the continent of Africa, 22 were represented in this body of literature, with Kenya and Ethiopia predominating. Figure 2(b) illustrates the massive geographic gap in this research, with Central African countries completely neglected, and Northern African countries also underrepresented.

Small-scale, sedentary (i.e. do not practice transhumance) livestock farmers were most widely documented (56 articles). Cattle were the most commonly mentioned livestock species (in 115 articles), followed by goats (101) and sheep (91; table SI.7), although most farmers kept more than one species. A considerable number of studies (39) did not mention the species of livestock being kept. Few publications focussed on responses of commercial farmers to climate change (figure 2(c)), with almost none focussing on large (industrial)-scale farms.

### 3.2. Characteristics and description of the responses

The 186 articles described 1089 responses of livestock farmers to climate-related hazards.

#### 3.2.1. Climate-related drivers of change

**Figure 3.**
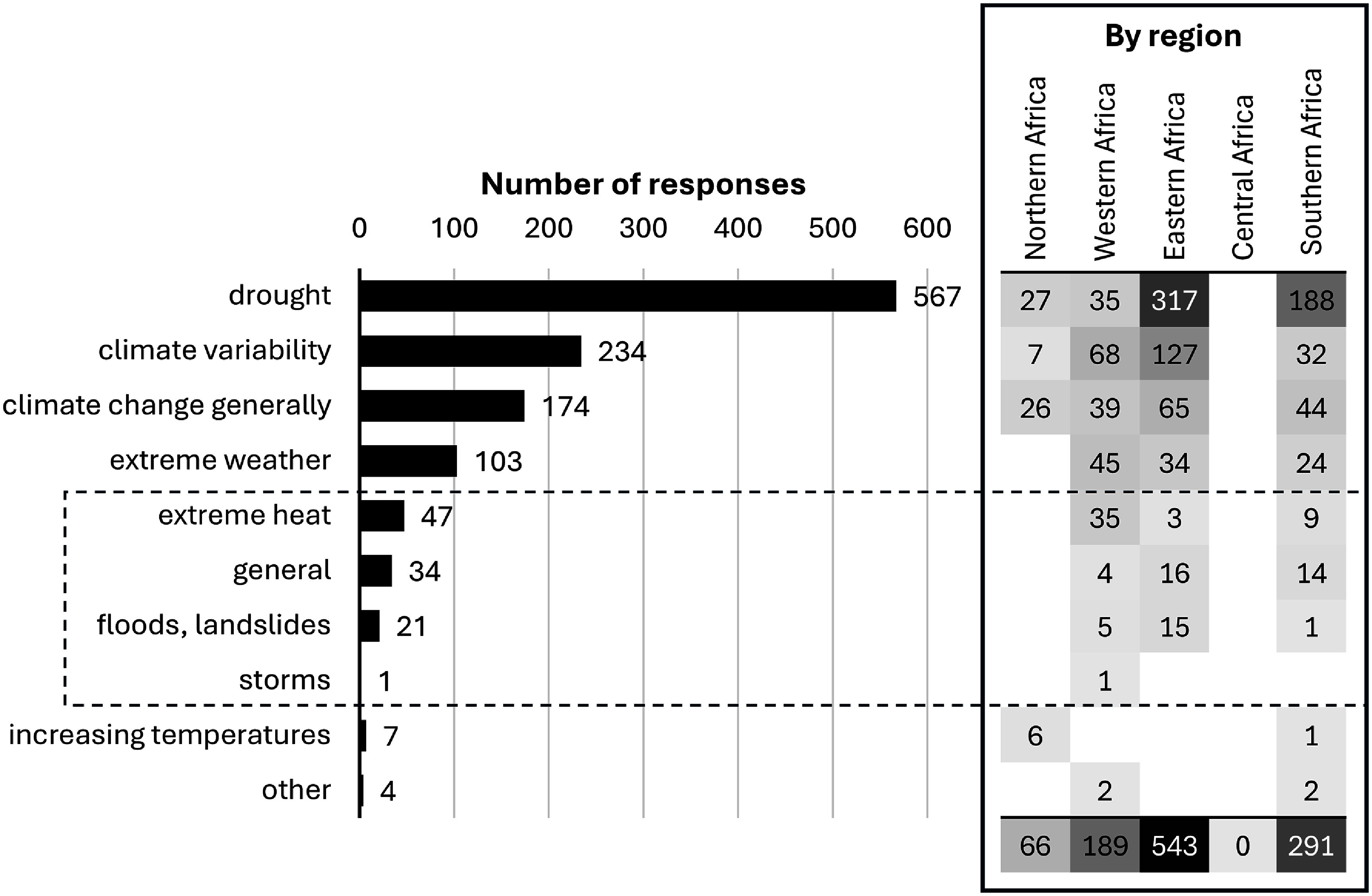
Number of times the different climate drivers were mentioned in relation to African livestock farmers’ responses, in total and by region (inset, right). Types of extreme weather are enclosed with the dashed box. ‘Other’ refers to articles that mentioned combinations such as heat and drought, or windstorms leading to wildfires.

Over half of responses were implemented in response to drought (figure 3), while a fifth were attributable to climate variability (usually variable or unpredictable onset or end of rainy season). This pattern remained when direct climate drivers were broken down by region, although heat was relatively more important to Western African livestock farmers than those based elsewhere.

**Figure 4.**
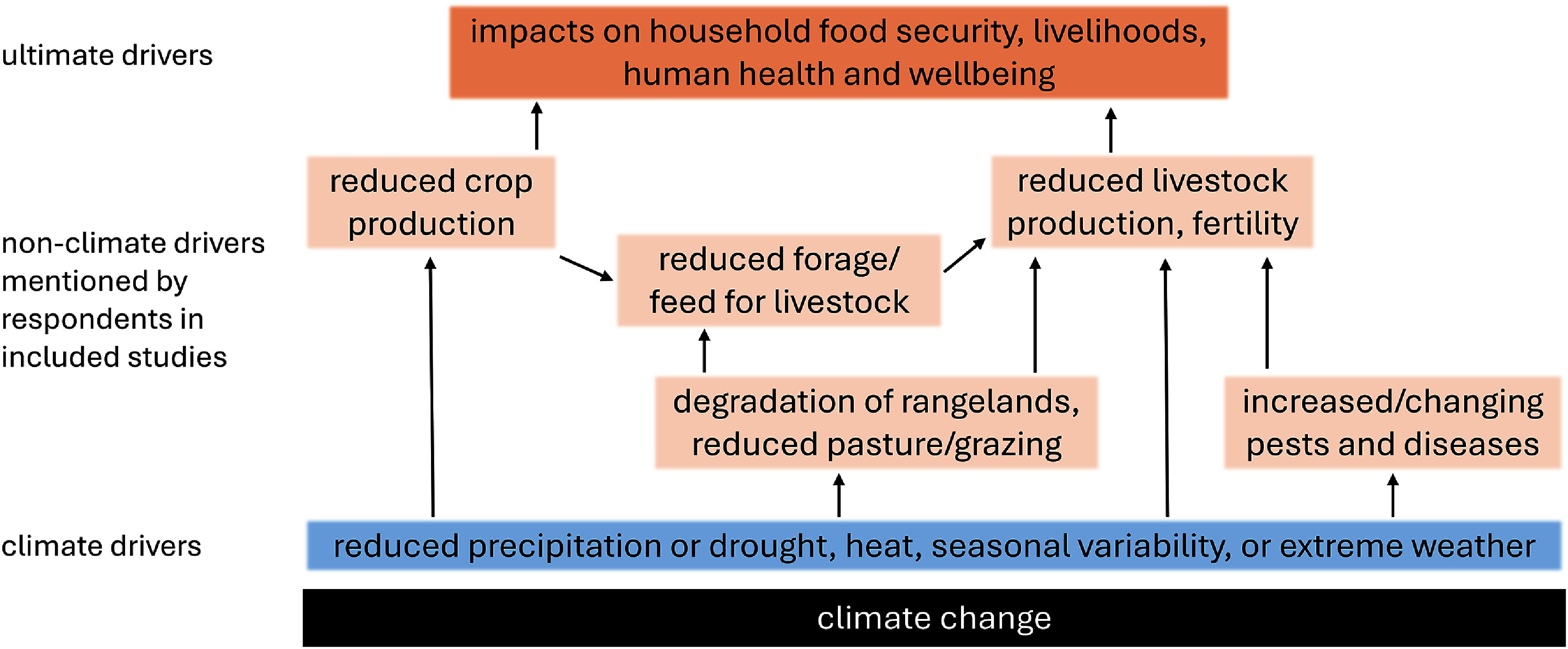
Conceptual illustration of the direct and indirect drivers of farmers’ responses: drivers secondary to climatic changes (in orange, referred to as non-climate drivers in this manuscript) and their relationship to the underlying/direct climate drivers (blue) that are projected to worsen as a result of climate change.

In about half the cases (566), drivers secondary to climate-related hazards were cited as the direct motivation to implement changes in management or behaviour (figure 4). The most commonly mentioned of these secondary, non-climate drivers were a shortage of feed (or pasture) or water for livestock (301 or 28% of responses), increased livestock pests or diseases (86, 8%), and the degradation of rangelands, including bush encroachment (83, 8%; see table SI.8 for a full list, and examples).

#### 3.2.2. Actors

Almost all (96%) of the responses were implemented by individuals (farmers or households, *n* = 1041), with the remainder by communities (18), government departments (20), and non-governmental organisations (NGOs) (5). There was also evidence of partnerships amongst actors, with government departments working with NGOs and faith-based organisations (11), and NGOs working with communities (9) to respond to climate-related hazards. While many of the responses implemented by government and NGOs related to the provision of aid or relief in response to disasters, the NGO-community partnership work tended to represent adaptation to reduce future vulnerability, such as the provision of disaster risk-reduction education, drilling boreholes, and the promotion of water harvesting and storage methods and home gardens.

Three-quarters of responses (801) were implemented by small-scale or subsistence farmers, and 164 by commercial farmers, with the scale of 167 responses unknown. Overall, a quarter of responses (276) were implemented by those who practice some form of transhumance. Finally, land tenure was not consistently described: a third (373) of responses were by those with their own land, a further third (362) by those on communal lands, and for the final third (354) tenure was not mentioned.

#### 3.2.3. Response categories and typologies

Over half (589) of the responses were implemented in advance of the climate-related challenges, with the remainder (500) implemented reactively. The desired outcome was mentioned for 285 responses, and mostly revolved around reducing the impacts of climate hazards (e.g. interventions that maintain or improve production, or reduce the incidence of livestock disease or mortality), or the vulnerability of their system to these hazards, for example, by selecting more tolerant breeds or species of livestock or diversifying household income. Ensuring access to- or conserving essential resources like water, livestock feed, or cash, were other desired outcomes.

**Figure 5.**
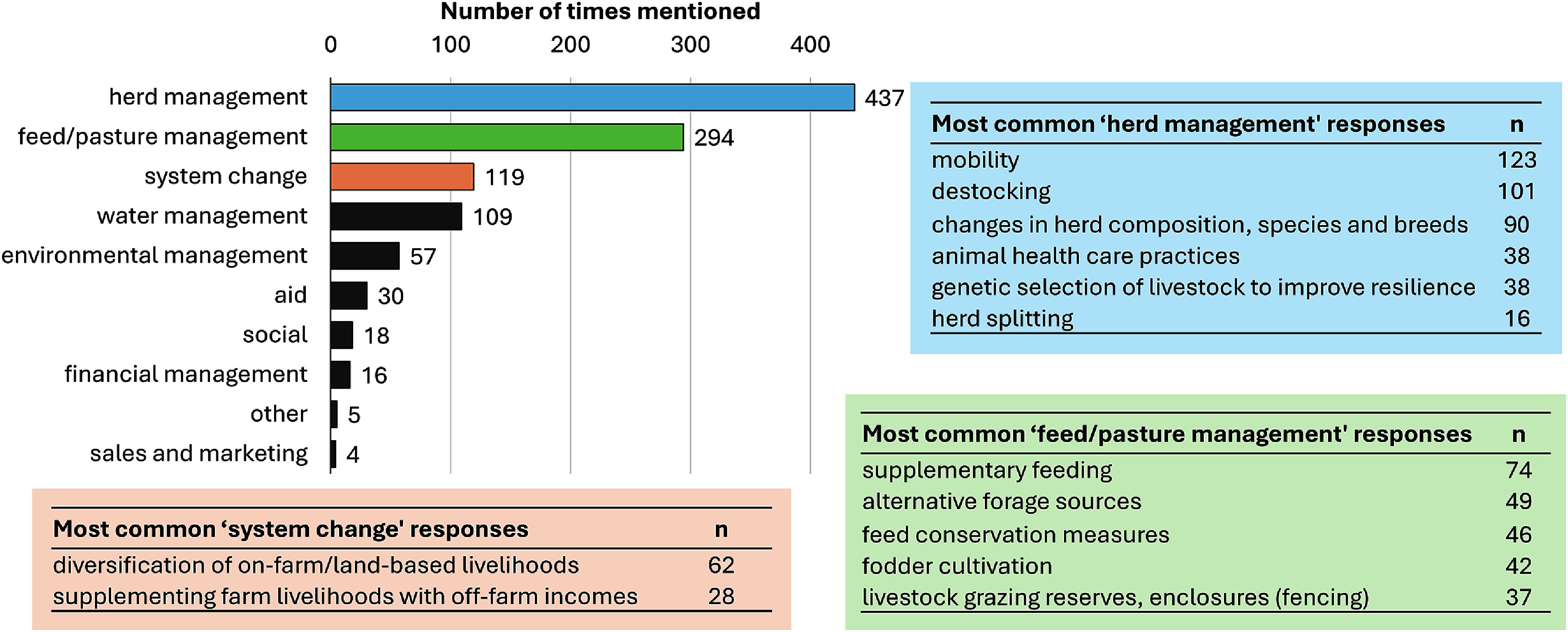
Overview of the main categories of responses applied by African livestock farmers, including examples of the most common responses (mentioned more than 10 times) for each of the top three categories (table insets).

There were nine main categories of adaptation response (figure 5; a full breakdown of these response categories is provided in table SI.9).

**Figure 6.**
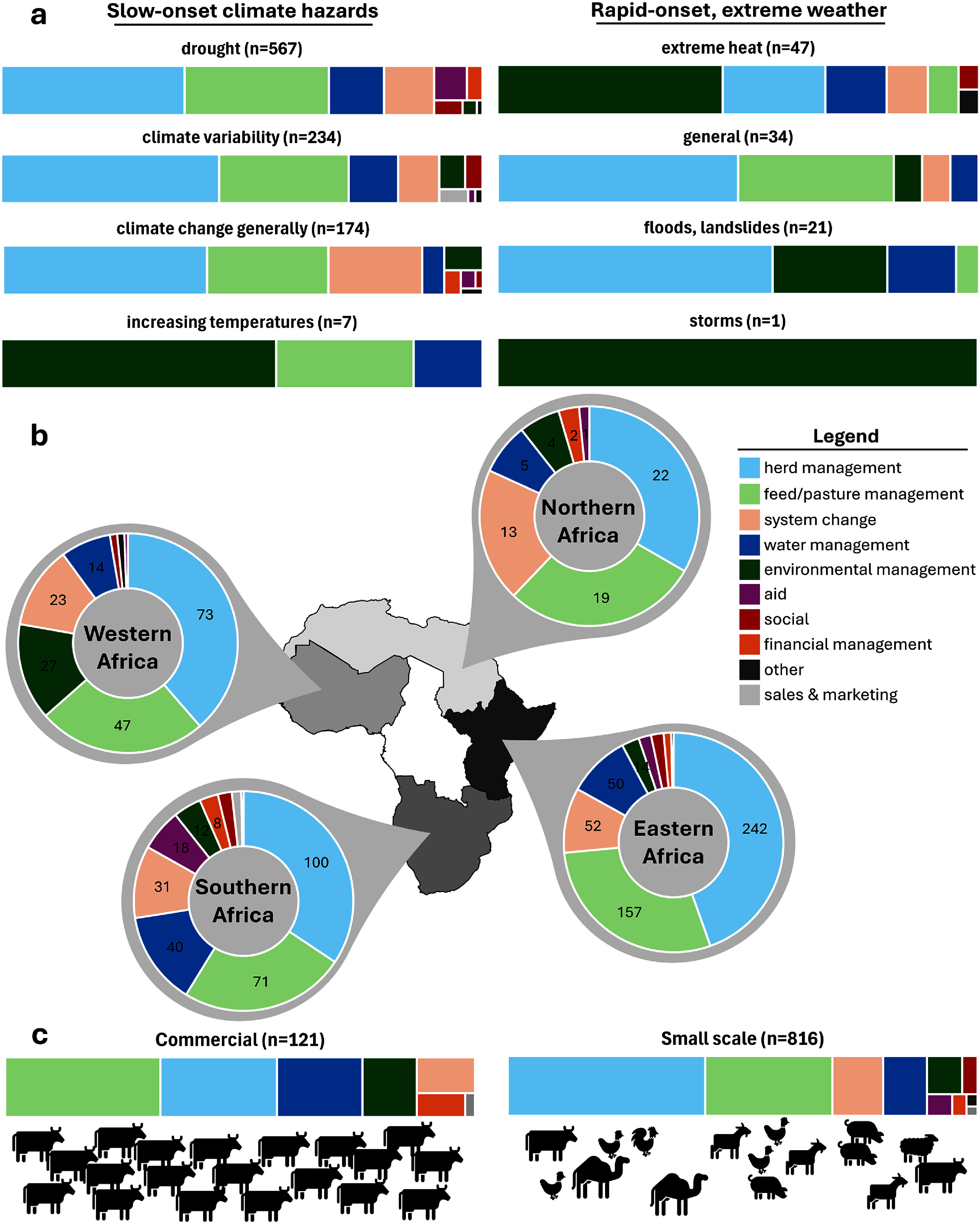
Distribution of response categories by climate driver (a), African region (b), and scale of farmer (c). African regions shaded according to the total number of responses from each (darker = more responses). Coloured spaces represent proportions of responses in each category, numbers in (b) represent the number of responses making up each segment. Results presented as percentages by row in table SI.10.

The responses varied by climate driver, region, and farming system (figure 6 and table SI.10). Across all categories, herd management responses were most frequently reported, followed by feed or pasture management. Most responses were reported from Eastern African countries, by small-scale farmers, and in response to drought. Aid—typically from government or NGOs, and in the form of feed for livestock, although occasionally as grants, water distribution or multidimensional—was only provided in response to drought. Farmers tended to implement environmental management in response to extreme heat (and, to a lesser extent, increasing temperatures and flooding), rather than other climate hazards, and was noted more in Western Africa than elsewhere. A greater proportion of Northern African farmers responded by changing their livelihood systems than from the other regions. Commercial farmers tended to focus relatively more on feed/pasture and water management-related responses than herd management, when compared to small-scale farmers.

There were a number of examples of responses from the different categories that represent environmental stewardship that are worth highlighting. These included preserving areas for dry season or drought grazing using fences or by temporarily removing water sources (Abate 2016, Ng’ang’a *et al* 2016a, Mihiretu *et al* 2020, Ndiritu 2021, Lelamo *et al* 2022), and improved pasture management, which was achieved by implementing rotational or controlled grazing (Mare *et al* 2018, Muricho *et al* 2018), clearing encroaching bush or alien species (Abate 2016, de Vries 2019, Pili and Ncube 2022), or reseeding pastures with locally collected indigenous grasses (Mganga *et al* 2015). Other examples of adaptation that required a sense of stewardship or ownership of land included the cultivation of various fodder crops, including indigenous and drought-tolerant species (many examples, including (Wetende *et al* 2018, Abdou *et al* 2020, Bosekeng *et al* 2020, Djohy and Sounon Bouko 2021)) and the propagation of drought tolerant fodder trees (Balehegn *et al* 2015, Wetende *et al* 2018, Dube *et al* 2021).

Most (60% or 652) responses fell within Frayne *et al* (2012)’s ‘limiting damage and protection’, though a third (38% or 414) represented efforts towards building long-term resilience. Only 23 responses were explicitly aimed at recovery.

Thornton and Manasfi (2010)’s ‘adaptation process’ typology best explained the full range of African livestock farmers’ responses to climate-related stressors, with only nine not clearly fitting into any of the categories. A quarter (28% or 301) of responses involved intensification of management practices, followed by diversification (20% or 213). Rationing- (154), innovation- (140) and mobility-related responses (127) were also fairly frequently reported, with only few examples of pooling (67) or exchange (72).

#### 3.2.4. Barriers and enablers

Of the 186 articles, 87 (47%) described barriers and 60 (32%) described enabling conditions for the farmers’ responses. Because some articles reported barriers (or enablers) for individual responses, and others listed all barriers to responding in general, it is difficult to compare the relative importance of different barriers for different responses. Thus, while we have reported the number of responses where each of the barriers was reported, these frequencies at best provide an estimate of the relative importance of each of the barriers, and should be interpreted as such.

**Figure 7.**
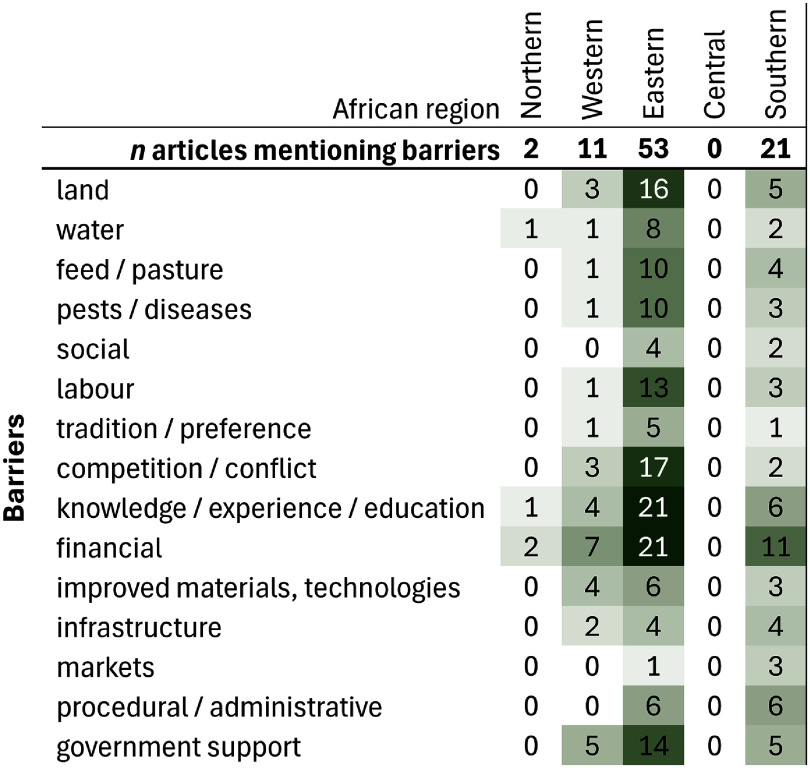
Barriers reported for the different African regions, with cell shading proportional to the number of times each barrier was reported.

A lack of money (or the cost of the response) was most commonly reported as being a barrier to adaptation, for all regions (figure 7). This manifested in several ways: the response may have been too expensive; farmers did not have the financial means to respond; or the response reduced productivity/income generation from the farm to the extent that it was not feasible. A lack of knowledge, including previous experience of climate hazards, traditional knowledge, own learning, and traditional ways of responding (Simotwo *et al* 2018, Wernersson 2018, Bosekeng *et al* 2020), as well as inadequate access to external sources of information, for example from government extension services or NGO projects (Chah *et al* 2018, Wetende *et al* 2018, Gebeyehu *et al* 2021), was highlighted as being a particularly common barrier in Eastern Africa. A lack of government support was noted as an important barrier in Western and Southern Africa. This included: prioritising crop agriculture over livestock; a lack of government policy on climate change; a lack of, or inadequate extension services; inadequate drought relief; and government settlement programmes, that do not consider pastoralist needs (Korbéogo 2014, Muller and Shackleton 2014, Wetende *et al* 2018, Ng’ang’a *et al* 2020, Anbacha and Kjosavik 2021, Lottering *et al* 2021). Access to agricultural inputs (e.g. feed, seed, medical treatments, improved genetics), infrastructure or equipment (including storage facilities and roads) (Chah *et al* 2018, Basupi *et al* 2019), and proximity to markets both for inputs and sale of livestock and products (Bosekeng *et al* 2020, Musara *et al* 2021), were also noted.

**Figure 8.**
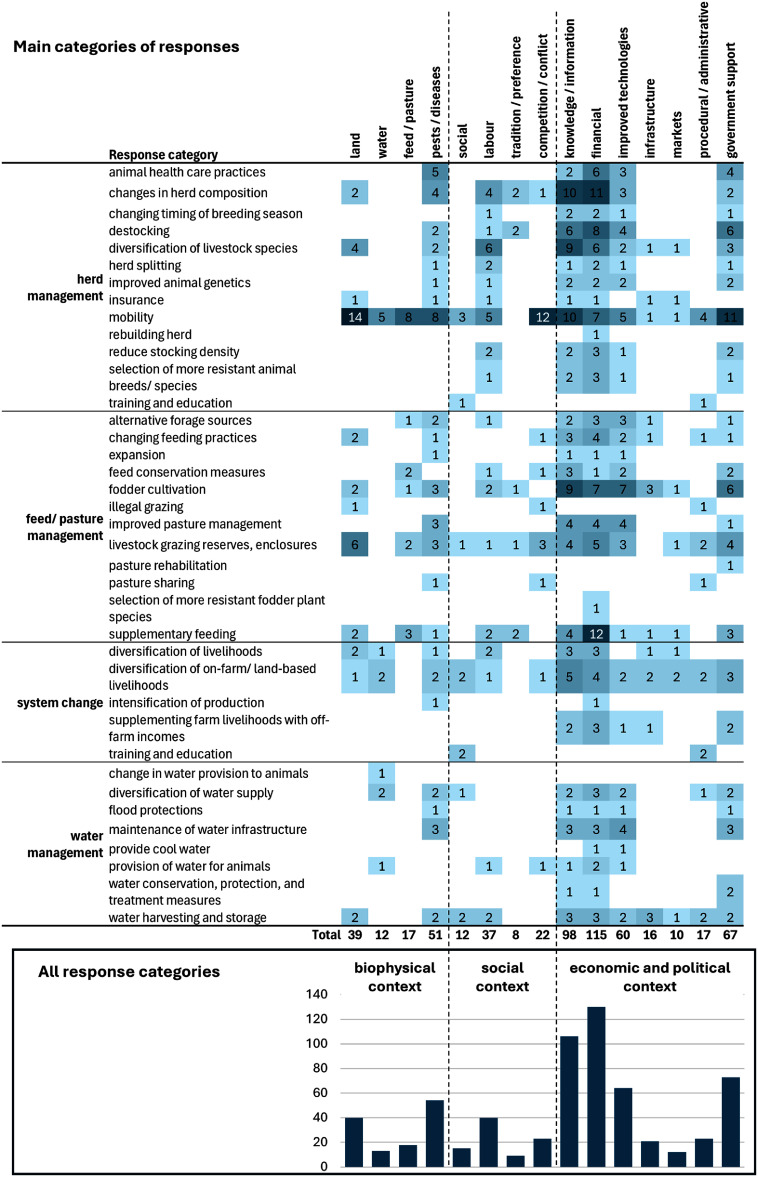
Barriers reported for the four main categories of responses (most would represent ‘a lack of’—the listed barrier). Main figure: heat map, with cell shading proportional to the number of times each barrier-response was reported; Inset figure: column chart of the total number of times each barrier was reported (for all response categories), with the barriers grouped by biophysical, social and economic/political contexts (Eisenack *et al* 2014). Lack of knowledge/information includes education and experience. The barriers for all categories of responses are presented in table SI.11.

Distinct patterns emerged when assessing which barriers were mentioned most frequently for each type of response (figure 8 and table SI.11). While money was mentioned frequently as a barrier overall, for the most commonly mentioned response, mobility, difficulties accessing land (particularly resulting from expanding areas of fenced-off croplands which obstructed livestock mobility routes (Byenkya *et al* 2014, Kgosikoma and Batisani 2014, Tumusiime *et al* 2018)), and competition and conflict (Korbéogo 2014, Camfield *et al* 2020) were the most common barriers. Knowledge and experience were highlighted as a major barrier to farmers changing the composition of their herd, or diversifying the livestock species they keep, particularly for pastoralists in Eastern Africa who were increasingly keeping camels but reported a lack of knowledge of camel husbandry practices or diseases (Boru *et al* 2014, Megersa *et al* 2014, Volpato and King 2019).

**Figure 9.**
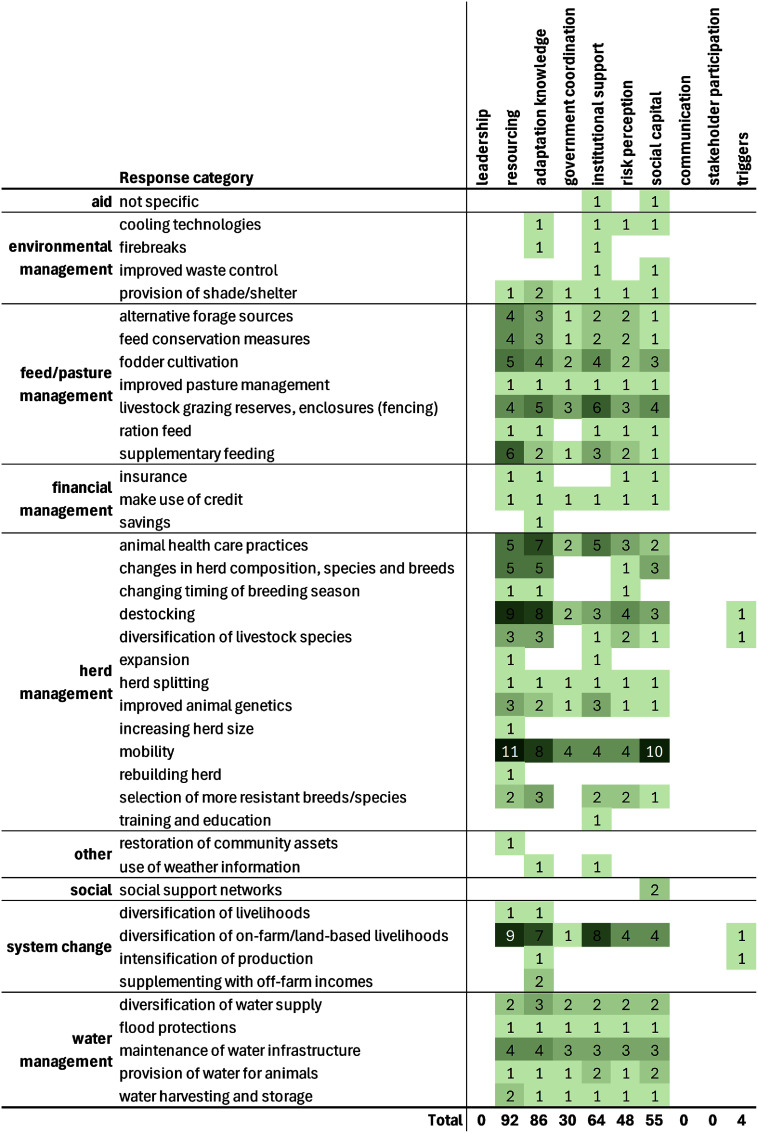
Factors that enabled farmers’ responses, for each response category. Categories of enablers modified from Brullo *et al* (2024): ‘resourcing’ refers to sufficient resourcing, and included having access to sufficient financial, infrastructural (e.g. markets), natural resources (water, feed, land) or labour; ‘social capital’ includes social networks; ‘communication’ refers to effective communication; and ‘triggers’ refers to triggering events or windows of opportunity for adaptation.

The most important factors enabling farmers’ responses were availability of and access to resources (money, feed or grazing and water for livestock, land, and labour), having knowledge of response options and how to implement them, and having sufficient institutional support to respond (including support of traditional institutions that regulate access to resources, and formal institutions that provide aid, information or services; figure 9).

## 4. Discussion

### 4.1. The literature

The dominance of African-led research in this field—a contrast to the findings of other similar Africa-focused reviews (Pasgaard *et al* 2015, Hunter *et al* 2020, North *et al* 2020)—suggests that there is a lot of local expertise and interest in the intersection of climate change and livestock, bringing this highly relevant topic for Africa into the peer-review realm.

While, in some ways, this was a robust body of information to draw on when analysing the detail of farmers’ responses, it also demonstrated some limitations. The literature describing African livestock farmers’ responses to climate challenges was skewed, both geographically, and on the topics covered. There was a critical lack of literature from Central Africa, with very few publications from Northern African countries, a gap also highlighted in the IPCC’s Sixth Assessment (Trisos *et al* 2022). These regions already suffer from the impacts of climate change, as well as social inequality, weak institutional capacity, and political unrest that compound their vulnerability (Birkmann *et al* 2022, Trisos *et al* 2022), and more research is needed to highlight feasible and effective options to improve their resilience to environmental and socioeconomic shocks.

The lack of reported research ethics oversight of these studies is concerning, although the recent improvement may reflect improving awareness of research ethics, and an evolving research culture. Funders, journals, and universities (including research supervisors) have a duty to promote—or enforce—research integrity (including ethical clearance), to ensure that research is well-designed, neither harms nor exploits participants for scientific purposes without feedback or perceptible benefits, and that it is capable of fulfilling its potential to benefit society (Gelling 1999, Horn 2017, North *et al* 2022a, Pizzolato and Dierickx 2023). The relatively limited funding available to African researchers compared to the rest of the world (North *et al* 2020) makes it even more critical that the research that is done, is done well.

The effectiveness of adaptation to climate change has been shown to be largely determined by the local context (social, economic, biophysical and governance), interacting stressors (climate and non-climate drivers) and barriers, the actors, and the system being acted upon (Moser and Ekstrom 2010, Thornton and Manasfi 2010, Eriksen *et al* 2011). Consequently, studies that do not report sufficient contextual detail undermine the value of their findings for broader assessments and application. Most of the included articles did not report these additional details—for example, many were not clear about how responses related to the climate driver—in terms of timing, what other drivers may have contributed to decisions, or desired outcomes. Few articles presented their results in a way that extends the field, or used recognised classifications (e.g. grazing system (FAO 1980, Simpkin *et al* 2020)), although some details could be inferred (e.g. farmer scale). Questions around barriers, enablers, drivers, or outcomes were not commonly asked or the answers reported. Some articles did not even report the species of livestock being kept, and most did not regard poultry as livestock, meaning that poultry was disregarded during interviews and reporting (which may account for the low incidence of poultry keeping-related responses). Considering the importance of poultry or village chickens for supporting household food security (Queenan *et al* 2016, Bettridge *et al* 2018, Mseleku *et al* 2023, Tenza *et al* 2024), this omission is a crucial gap. Future research should provide more information on these aspects.

Many of these gaps could have been addressed during the study design phase if authors from different academic disciplines, across the natural and social sciences, and from policy and practice, were included in the research team. In fact, with ‘wicked’ or intractable cross-cutting challenges, which the deeply complex relationship between climate change and society epitomises (Termeer *et al* 2013, Sun and Yang 2016), a widely advocated approach is an inter- or transdisciplinary one, in which diverse research teams pool and integrate multiple perspectives (Swart *et al* 2014, Victor 2015, Degroot *et al* 2021, Hunter *et al* 2022, Rising *et al* 2022, North *et al* 2022b).

### 4.2. The responses

The predominance of responses to drought and climate variability is unsurprising, given the magnitude and geographic scale of impact these hazards have on agriculture, particularly in arid or semi-arid regions that rely predominantly on natural rainfall (You *et al* 2011). However, with the increasing incidence of severe heat globally (Giguere *et al* 2025, Martinez-Villalobos *et al* 2025) and in Africa (Ranasinghe *et al* 2021, Trisos *et al* 2022), and what is known about the erosive impacts of heat on livestock health and productivity (Thornton *et al* 2021, North *et al* 2023), more research attention needs to be paid to the impacts of, and responses to, heat stress.

Unlike similar reviews of adaptation (Berrang-Ford *et al* 2011, Hunter *et al* 2020), where low-income countries tend to be characterised by reactive adaptations, this study found that more than half of the responses were implemented in anticipation of the hazard. This may reflect the frequency with which livestock farming is affected by such hazards, and consequently, the wealth of local and traditional knowledge and mechanisms for responding to these recurring challenges (Niang *et al* 2014).

The lack of research on commercial farmers’ responses was unexpected, even accounting for the predominance of small-scale farmers in Africa (Herrero *et al* 2017, Lowder *et al* 2019), when one considers the long-established discipline of animal science, and the fact that commercial production is highly data-driven (Rodriguez-Ledesma *et al* 2015). However, animal science research tends to retain a heavily discipline-specific focus on improving production efficiencies (Rodriguez-Ledesma *et al* 2015), and, consequently, might not make use of key terms relating to climate change, or adaptation. Moreover, the results of experimental research were excluded from this review, which may have selected against animal science research. Finally, the data underlying modern, large, intensive commercial production, may be held within industry, or might derive from work conducted in the Global North, rather than Africa, as was observed in a meta-analysis of heat stress research (North *et al* 2023).

Nevertheless, the relatively poor coverage of commercial farming in the climate change adaptation literature is concerning. Commercial farming, with its market-focus and efficiency-driven production, contributes to national food security and GDP, as well as food security in neighbouring countries and internationally via exports (Olbrich *et al* 2013, Temoso *et al* 2016, 2024, DALRRD 2024). It also supplies food to rapidly urbanising areas (Mirzabaev *et al* 2019), and contributes to employment (Christiaensen and Maertens 2022). While commercial farms may not be as immediately vulnerable to climate change as limited-resource, small-scale producers, since they often have greater assets, market access, and institutional support (Godde *et al* 2021), any impacts to these farming systems, resulting in loss of production or even the decision to step away from farming altogether, has the potential for large-scale repercussions (Tibesigwa *et al* 2017). A lack of research into and communication of African commercial farmers’ climate-related challenges and the mechanisms they employ to mitigate risk results in a bias in the literature, and subsequently biased assessments and recommendations filtering through IPCC and FAO reports, and thereby in government priorities and responses to climate-related disasters (Mare *et al* 2018, Lowder *et al* 2019). Even with this imbalanced sample, it was clear that commercial and small-scale farmers tend to focus on managing different aspects of their systems to reduce risk. Better understanding of the different needs and priorities of small-scale vs commercial farmers to reduce their vulnerability to climate-related shocks would support informed decision-making at the national level—for example, what support would be most beneficial to whom during climate-related disasters. Moreover, a clearer understanding of the different needs and strategies of different types of farmers could inform targeted knowledge transfer efforts, for example through extension services, providing tools appropriate for improving the sustainability of their specific production systems, under a future with climate change.

While most responses tended to be incremental changes to herd management that allowed farmers to ‘get by’, a notable portion represented transformation of farmers’ livelihood systems (for example, by changing from cattle to camels, or by diversifying their sources of income), or anticipatory actions to reduce vulnerability (e.g. conservation of grazing or fodder). This is indicative of bottom-up, proactive adaptation, where, in the absence of government interventions, farmers are solving their problems using innovative, localised solutions. Governments need to recognise and acknowledge these developments, and ensure that any shifts in policy or practice promote effective local practices, rather than stifling them (Brown *et al* 2017, Conway *et al* 2019).

With the expansion of the adaptation literature has come many proposed systems of classifying adaptation responses. These include those proposed or adapted by Berrang-Ford *et al* (2011), Biagini *et al* (2014) and Lesnikowski *et al* (2015) for assessing adaptation globally. However, when applied to the responses in this review, primarily on-farm decisions and motivations of livestock farmers, typologies by Frayne *et al* (2012) and Thornton and Manasfi (2010) proved the most useful and able to capture the range of response types and objectives. The process typology of Thornton and Manasfi (2010) (mobility, exchange, rationing, pooling, diversification, intensification, innovation, revitalisation) and the asset-adaptation lens used by Frayne *et al* (2012), both map directly to the large majority of herd-management, feed/pasture, and livelihood diversification responses captured in our review. Similar to the findings of Hunter *et al* (2020), many of the global adaptation typologies were unable to accommodate most of the responses, either being too broad to provide much insight into what farmers were doing (for example, Epule *et al* (2017)), or having a Global North, top-down focus, and therefore not relevant to the actions taken by African farmers on the ground (Berrang-Ford *et al* 2011, 2014, Biagini *et al* 2014). Meta-level tracking approaches (outcome-based, readiness/proxy, and policy/program inventories) are useful for national-level monitoring but are typically too coarse to capture farmer-level strategies (Ford *et al* 2013, Berrang-Ford *et al* 2014). Likewise, typologies derived from donor/project documents (Biagini *et al* 2014) are valuable when translating farmer actions into policy or funding language (capacity building, physical infrastructure, early warning), but they tend to underrepresent informal, autonomous tactics.

Across the board, most of the barriers to adaptation that were mentioned by livestock farmers related to insufficient resources (financial, information/knowledge/skills, and infrastructure). While barriers frequently co-occur, making it harder to successfully adapt to stressors (Simpkin *et al* 2020), the ways barriers were reported in the publications made it impossible to assess co-occurrence in this review. As noted by Eisenack *et al* (2014), barriers to different types of responses varied, with specific resources or social factors constraining some responses (like mobility), and access to information constraining others (particularly when the response involved ‘trying something new’, like taking on new livestock or fodder plant species). Since most of the included articles were based on interviews with farmers, it is not surprising that the focus was largely on the most immediately obvious ‘factors that make it harder to plan and implement adaptation actions or that restrict options’ (Klein *et al* 2014, p 907), like a lack of information or resources, rather than higher level barriers such as lack of political capacity or will (Nkuba *et al* 2021). A clearer picture of all the factors impeding adaptation by African livestock farmers would require more nuanced case studies that tease apart the obstacles and how they are affected by the specific local context, actors, and system (Moser and Ekstrom 2010).

Unsurprisingly, many of the enablers mentioned in the included articles echoed Brullo *et al* (2024)’s findings relating to enablers for agricultural households, including: access to sufficient resources (natural, social, financial, physical and human); having sufficient knowledge (traditional or scientific) to perceive the risk and thus the need to respond, and of the different response options available; and guidance or oversight from responsible institutions. These largely align with the concept of adaptive capacity, or the ‘biophysical and socioeconomic factors that enhance human capability to adapt to and recover from adverse impacts of climate change’, representing ‘a system’s ability to mobilise resources in response to stresses and shocks’ (Chapagain *et al* 2025, p 2). However, as with the barriers, missing from the included articles were mention of the higher-level enablers, such as proactive leadership, coordination of different levels of government, effective communication, and participation of communities and other stakeholders in decision-making around adaptation initiatives (Brullo *et al* 2024), as well as the agency of the livestock farmers themselves to adapt (Chapagain *et al* 2025).

Reducing the vulnerability of Africa’s livestock farmers requires the systemic barriers inhibiting their responses to be addressed, putting into place interventions that improve the agency and flexibility of the farmers, and that create an enabling environment. By knowing which are the greatest barriers to adaptation, particularly which barriers relate to which kinds of adaptations, governments and NGOs can ensure that adaptation programmes include mechanisms for addressing these barriers. Crucially, while addressing barriers would make some responses more feasible, Brullo *et al* (2024) note that creating an enabling environment for adaptation requires a combination of interrelated enabling conditions (for example, sufficient resources, and knowledge of adaptation options, and institutional support), rather than an exclusive focus on removing barriers.

While many of the enablers were localised and internal, examples of impactful interventions that could be scaled up included: the provision of information and early warnings (Ndiritu 2021, Maina *et al* 2022); training on response options and skills to support responses (Opiyo *et al* 2015, Abdou *et al* 2020, Snaibi *et al* 2021); support services like farmer cooperatives, agricultural extension and animal health care services (de Vries 2019, Liverpool-Tasie *et al* 2019, Fanadzo *et al* 2021); infrastructure like roads, storage facilities (livestock feed and water) (Fanadzo *et al* 2021); and access to markets (Zampaligré *et al* 2014, Opiyo *et al* 2015, de Vries 2019). Access to finances though credit, subsidising certain actions (like timely destocking or various insurance options), and supporting savings groups, were also important predictors of whether farmers could respond or not (Ng’ang’a *et al* 2016b, Asayehegn *et al* 2017); although these interventions all need to be carefully designed and implemented to improve long-term rural resilience.

The results of this systematic review point to possible areas of intervention by governments, NGOs and individual farmers across Africa, where top-down interventions can be implemented to complement or enhance what African farmers are already doing on the ground (Brown *et al* 2017). More government interventions are needed to support farmers, by addressing barriers and ensuring resources, including knowledge and skills (not just technical (de Lange *et al* 2025)), are available to enable proactive and informed management and decision-making to reduce the vulnerability of livestock farming to climate variability and change. Current collaborative efforts between NGOs, government, and communities to build resilience (e.g. as reported by Ndlovu *et al* (2020) and Abdou *et al* (2020)) could be scaled up, as this multi-level and multi-partner adaptation has been identified to be effective at building resilience elsewhere (Leck and Simon 2013, Lassa *et al* 2023).

## 5. Limitations

Because the information prescribed by this review was ground-level action, ‘case study’ style papers predominated. This means that large-scale (global or regional) modelling or synthesis work was excluded—and may be the explanation behind the primarily local authorship in this sample. It is also important to note that this review is an aggregation of the results of smaller studies whose authors already had to aggregate the results of their interviews, and so in the process much of the context, the nuance behind responses and the consequences of responses may have been lost. While this review only recorded responses that were directly or indirectly linked to climate, we acknowledge that there are many drivers of change, including social, economic and political pressures (Simpkin *et al* 2020). Many of these drivers will be interacting with the climate-related drivers to push livestock farmers to adapt their systems. The body of literature drawn upon ends at the end of 2022, but due to resource constraints, it was not possible to extend this to closer to the publication date.

## 6. Recommendations

The distinction between crop and livestock farming is artificial for most African farmers, with most keeping a mixture of the two depending on available resources (Thornton and Herrero 2015). While responses aimed at adapting livestock systems in isolation may be appropriate for intensive, commercial enterprises, for the majority of African farmers, livestock form part of a diverse portfolio of cultural, income- and food-generating activities (Pell 1999, Waha *et al* 2018). The purpose of adaptation for these farmers is to maintain the stability of the system as a whole, and thus their livelihood and household food security, with different elements being used to counterbalance impacts on others: responses that only address the risks to livestock does not make sense or address the needs of these farmers (Garbole *et al* 2025). Systems approaches that consider livestock as an element of the wider livelihood system and environment, and evaluate responses for their potential to reduce risk to the system as a whole in a sustainable way (Bilotto *et al* 2024), could be employed both by academics in future adaptation research, and practitioners evaluating different options for implementation.

We propose a number of specific recommendations for future research. These include improved research integrity and ethical oversight training and support from institutions, and more stringent ethical requirements from funders and journals. We also call for research that: addresses geographic and topical gaps; better describes the contexts in which farmers are adapting; considers non-traditional animal species being kept for livelihoods; reports the primary purpose of the livestock and notes whether the system is intensive/specialised, or mixed/diversified; evaluates and reports the longer-term outcomes of different responses; or investigates the barriers to preferred responses at different scales. We also recommend the use of a wider selection of research methods tailored to and appropriate for the specific research population and questions, including quantitative methods to model changes in livestock production yields in response to different adaptation approaches (Escarcha *et al* 2018); inter- and transdisciplinary action research that includes more depth and has policy and action relevant outcomes (e.g. Froebrich *et al* 2020); or makes use of systems approaches, to better capture the wide range of interlinked land-based livelihoods (including crop and livestock agriculture, harvesting wild foods and products, and bee keeping) and how rural people are diversifying their livelihoods to survive (e.g. Glendell *et al* 2025).

African farmers are responding to multiple stimuli, not just climate change (Adimassu and Kessler 2016). If adaptation responses have multiple co-benefits, farmers are more likely to withstand different types of stressors, that include climate shocks, but also economic, political and other stressors (Berrang-Ford *et al* 2011). Therefore, when planning adaptation projects, practitioners and governments should evaluate the relative benefits of different responses for reducing vulnerability, or improving the resilience and sustainability of land-based livelihood systems to a range of possible stressors (Osbahr *et al* 2010). Some examples of responses that improve resilience include those that diversify livelihoods, by including different forms of production on the same land (e.g. mixed species herds, mixed farming, bee keeping, agroforestry) (Mganga *et al* 2015, Opiyo *et al* 2015, Fadina and Barjolle 2018, Muricho *et al* 2018), or incorporate off-farm activities with land-based incomes (Mayanja *et al* 2020, Ndlovu *et al* 2020). Researchers can support this by providing data to support different livelihood combinations in different contexts (see for instance Bostedt *et al* (2023)), and how they respond to different stressors, or their vulnerability to different climate scenarios (Stadtbäumer *et al* 2022).

Governments should consider the main barriers that were highlighted by the study and put in place interventions to reduce them. Among these include improved sources of information for farmers to support better decision-making—by improving resources allocated to the departments overseeing agriculture and extension, to increase farmers’ access to extension services (Ayim *et al* 2022). Complementing greater access to extension, educators at tertiary institutions need to update curricula to ensure that the message of climate variability and change, and adaptation options, is sufficiently emphasised, particularly for agricultural extension agents and similar outreach workers who have regular and trusted contact with farmers (Belay *et al* 2024).

Governments should ensure that policies support and do not undermine traditional institutions that manage access to, and use of, land and other natural resources (Basupi *et al* 2017). Improved public infrastructure, like roads (World Bank 2024a, 2024b), which facilitate access to markets and agricultural inputs, and wide communication of early warning of climate-related hazards (Pienaah *et al* 2023), are all crucial for improving farmers’ ability to respond to stressors.

## 7. Conclusions

During this review, a range of different actions that African livestock farmers are implementing in response to climate variability and change were recorded from the literature, predominantly relating to herd, fodder, and water management. Within these broad categories a wide range of responses were observed. Despite a lack of institutional support and limited access to resources, in general, African farmers demonstrate a good awareness of climate hazards, and implement responses that allow withstand climate-related impacts. However, a large proportion of farmers are responding reactively, without information, planning or support. These farmers are likely to become progressively less able to withstand shocks, increasing food insecurity, rural-urban migration, and worsening social conditions.

While a sizeable body of literature describing African livestock farmers’ climate-related challenges and responses exists, this review highlights a number of issues that could be addressed to ensure future research better supports policy and planning. With resource constraints limiting African researchers’ capacity, it becomes even more important that investigations comply with ethical standards and are sufficiently rigorous for maximum social impact. Funders and research organisations need to ensure that research into adaptation responses and options is designed, implemented, and reported in such a way as to better address the needs of policymakers and other stakeholders, providing information that can inform implementation, as well as policy and planning at different levels of government. Governments have a responsibility to address barriers that are impeding adaptation to climate variability and change, to help farmers respond appropriately and sustainably, to improve livelihoods, food security, and better manage land for generations to come.

## Data Availability

All data that support the findings of this study are included within the article (and any supplementary files).
